# Eliminating the Glass Ceiling in Academic Psychiatry

**DOI:** 10.1007/s40596-017-0810-5

**Published:** 2017-11-06

**Authors:** Jeffrey A. Lieberman, Anke A. Ehrhardt, H. Blair Simpson, Melissa R. Arbuckle, Abby J. Fyer, Susan M. Essock

**Affiliations:** 1 Columbia University, New York, NY USA; 2 New York State Psychiatric Institute, New York, NY USA

## The Gender Gap in Academic Psychiatry

In academic medicine, as in many employment settings, women frequently earn less than men and hold fewer senior positions [1–5]. While the magnitude of this disparity varies across professions and employment sectors, it is virtually ubiquitous, and academic medicine is no exception. Among physicians with academic appointments at public medical schools in the USA, women are paid about 80% of what men are paid ($207,000 versus $258,000; data from 2011 to 2013) [1]. Gender disparities should be a concern for all medical specialties, but they are of greatest importance to those that attract the highest proportion of women trainees. Given the number of women pursuing training and careers in psychiatry, departments need to consider whether a gender gap exists among their faculty to improve the mental health workforce, quality of care, and financial and ethical reasons.

Gaps in pay or other metrics reflecting departmental investments by gender may reflect conscious or unconscious bias against women, hence examining data over time can help identify disparities and inform actions to address root causes, including gender bias [6, 7]. To this end, we examined our department’s performance by looking at the following domains by gender: academic rank, salary, leadership positions, and allocation of departmental resources. In addition, revenues generated through sponsored research and clinical services were compared for men and women faculty. As these results subsequently demonstrate, women are crucial to the economic health of the department, so, in addition to the ethical imperatives to promote fairness and increasing the quality of the science produced [8], it makes good economic sense to promote gender parity.

## Drilling Down on Departmental Data

Columbia University’s Department of Psychiatry (hereafter, Columbia Psychiatry) is comprised of Columbia University College of Physicians and Surgeons Department of Psychiatry, the Psychiatric Services of New York-Presbyterian Hospital-Columbia University Medical Center, and the New York State Psychiatric Institute. Columbia Psychiatry is one of the nation’s largest and leading academic psychiatry programs, with nearly 500 salaried faculty with primary appointments in psychiatry (215 men and 259 women). As such, our numbers are large enough to look at temporal trends with respect to gender parity. Thousands of clinicians, researchers, and educators have passed through Columbia Psychiatry’s ranks, with many more to come. Thus, we felt that it was essential to examine our practices to identify any apparent gender disparities and act to ensure that we practice the principles we espouse.

## Faculty Number and Academic Rank by Gender

Nationally, 38% of physicians at academic medical institutions are women. Women are well-represented in the lower academic ranks but under-represented at higher ranks, with roughly equal numbers of men and women Assistant Professors, a 3-to-2 men-to-women ratio at the Associate Professor level and 3-to-1 ratio for Full Professors [9].

**Fig. 1:**
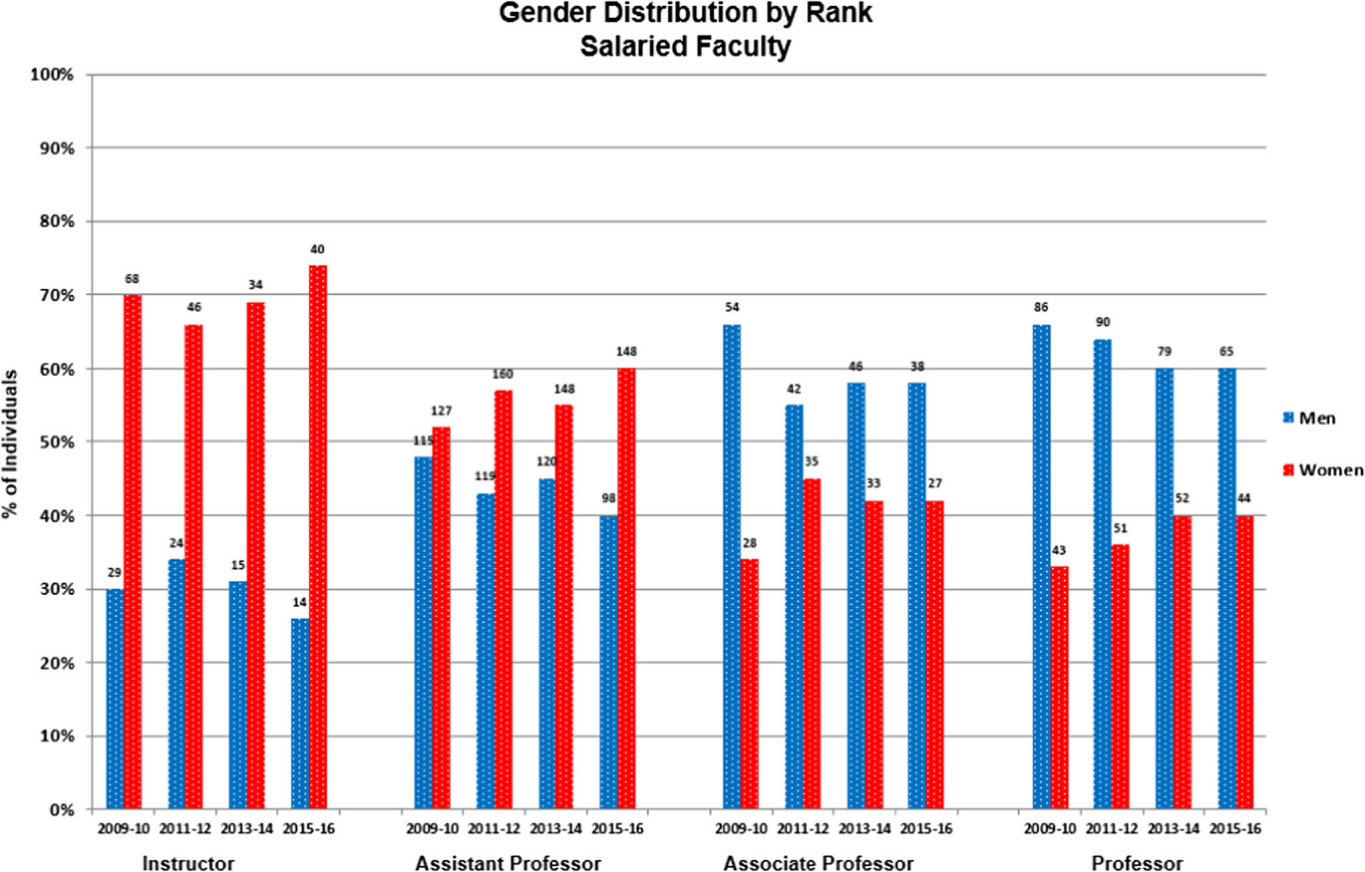
Gender distribution by rank over time at Columbia Psychiatry. Numbers atop bars reflect the number of salaried faculty at that rank at the close of the academic year whose primary academic appointment was in Psychiatry, regardless of percent time employed

Figure 1 shows the number of men and women salaried faculty with primary academic appointments in Columbia Psychiatry at each rank over a 6-year period. Consistent with national trends, women are under-represented in the more senior ranks of our department, though less so than nationally. Women comprised 63% of our junior faculty (instructors and assistant professors), but only 40% of professors. This indicates that we have comparable or greater numbers of women than men entering the pipeline, suggesting either greater attrition or lack of promotion of women faculty. Lack of promotion seems unlikely to account for the gap as, when we examined time-in-rank, at each rank, men tended to remain in the rank about 1 year longer than women. Thus, our challenges appear to lie more in retention—assuring a welcome, productive workplace with opportunities for career development for all faculty.

## Career Development and Leadership Positions

We surveyed gender equity in career development opportunities for our faculty. The leadership structure of our department consists of the chair, 6 vice chairs, and 20 division directors. Like many departments of psychiatry, Columbia has never had a woman chair. Currently, 4 of the 6 vice chairs are women, and 9 of the 20 division directors are women. Women faculty also head 3 of the department’s 5 inpatient units.

In addition, we studied whether faculty, in the course of their career development, had achieved sufficient national visibility to become successful candidates for senior positions elsewhere. In the past decade, 15 faculty members have been recruited to senior academic positions at other institutions including the NIMH directorship, 5 department chairs, and 1 vice chair position. Nine of the 15 were women, including 4 of the 5 individuals recruited to be department chairs and the 1 vice chair. Three of our faculty (2 of them women) were also appointed as Dean for Student Affairs and Associate Deans for Education.

## Salary

**Fig. 2:**
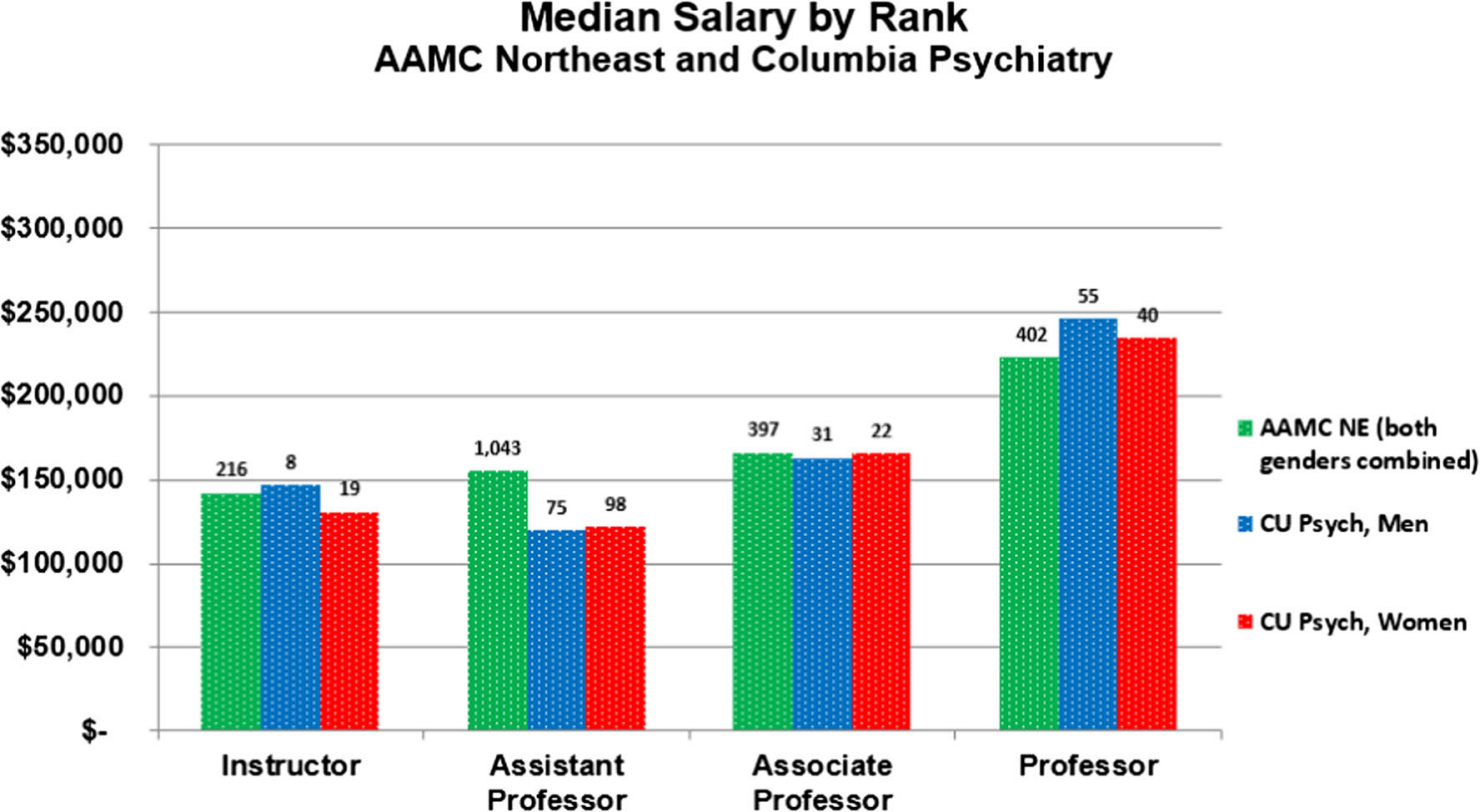
Median Salary by Rank 2015–2016 academic year, AAMC Northeast compared with men and women at Columbia Psychiatry. For individuals at Columbia working less than full time, salary used was the full-time equivalent. Includes psychiatrists and doctoral-level research scientists salaried at least 50% time in Psychiatry at any time during the academic year; excludes the department veterinarian and department Chair

Department funds support our academic mission; hence, how these resources are distributed is an indicator of how we value and invest in our faculty. Therefore, we examined total compensation for all Columbia Psychiatry doctoral level faculty (MD and equivalent and PhD and equivalent), grouped by rank and gender. We found that the median salaries for men and women were roughly comparable (Fig. 2). At the Assistant and Associate Professor levels, median salary for women exceeded that for men by $1999 and $2557, respectively (Fig. 2). At the Full Professor level, median salary for men exceeded that for women by $11,507, a gap of about 5%. The largest difference in salary ($15,093) was at the instructor rank (Fig. 2). At Columbia, the instructor rank is used much less often than the other ranks, and the people holding this rank often fill specialty niches within the department, as reflected in the overall-higher salaries for instructors than assistant professors.

We used the data from the Association of American Medical Colleges (AAMC) Northeast region as a benchmark with which to compare our faculty salaries [10] (Fig. 2). Since the annual AAMC Faculty Salary Report does not specify salaries by gender, the data displayed in Fig. 2 show the AAMC data of Northeast medical schools for both genders. At the Professor rank, our faculty are paid an average of about $25,000 more per person than other programs in the Northeast, (some of which may be attributable to the high cost of living in New York City) while salaries were lower for both men and women Assistant Professors.

**Fig. 3:**
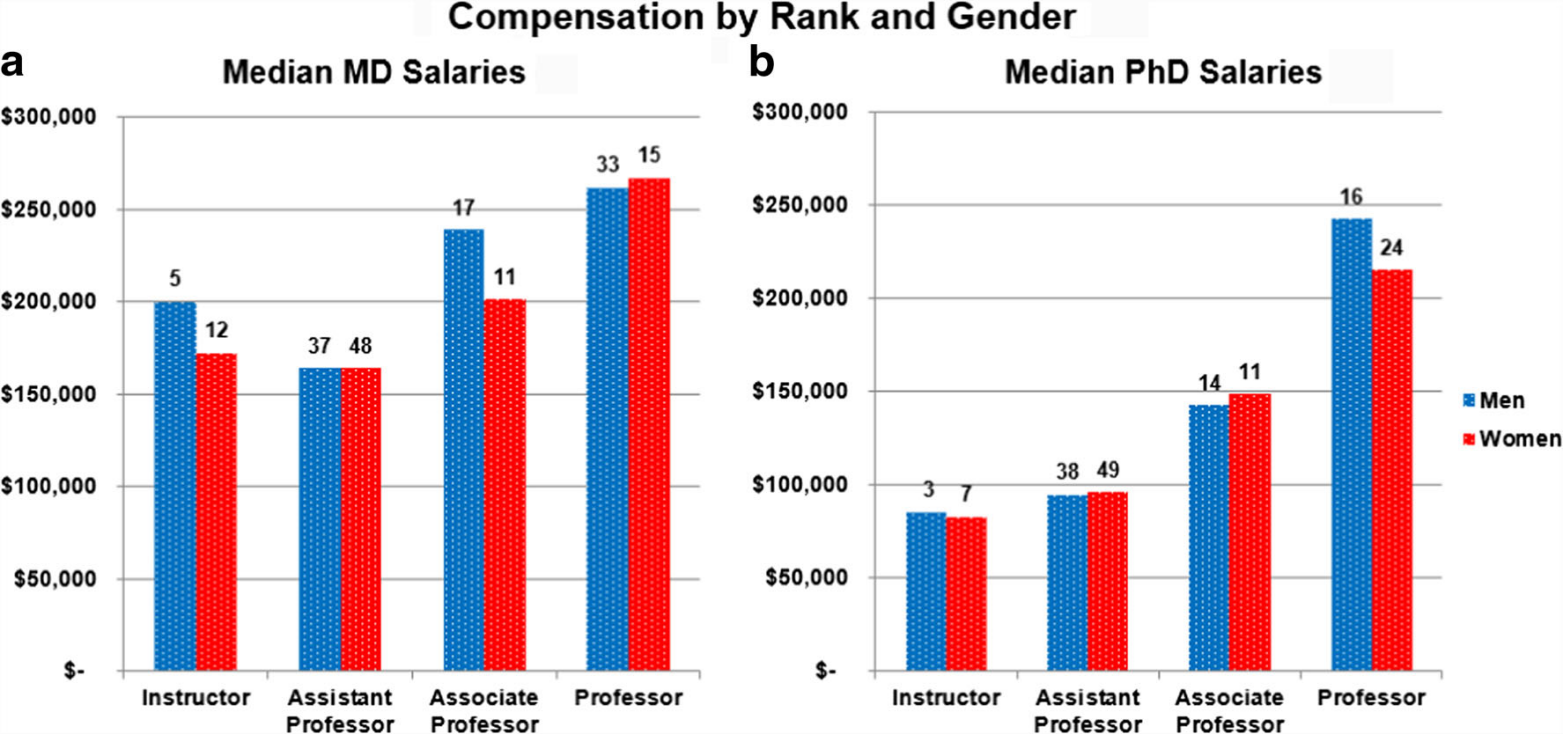
**a** Median Salary by Rank and Gender, psychiatrists (including individuals holding MD PhD degrees) with academic appointments receiving salary from Columbia Psychiatry. For individuals working less than full time, salary used was the full-time equivalent; excludes department Chair. **b** Median Salary by Rank and Gender, non-physician doctoral level faculty in Columbia Psychiatry. For individuals working less than full time, salary used was the full-time equivalent. Includes doctoral-level research scientists except the department veterinarian. Faculty holding combined MD PhD degrees are included with MDs in Fig. 3a

Because physicians and other doctoral-level faculty may differ in salaries, especially at the junior ranks, we compared salaries of psychiatrists, including those holding both MD and PhD degrees (hereafter for brevity all called MDs), with other doctoral level faculty (hereafter, PhDs) in the department. Figure 3 shows the salary data for MDs and PhDs separately, illustrating that, as is also the case nationally [10], the salary gap between physicians and non-physicians decreases with seniority in academic rank. This narrowing in compensation suggests that, at the upper academic ranks, salary is predominantly determined by one’s productivity. Women MDs who are full professors in the department earn on average 102% that of their male colleagues (Fig. 3a) despite fewer years at that rank (means of 10.0 years for women, 14.2 years for men), while women PhDs who are full professors earn on average 89% of their male colleagues (Fig. 3b) despite a similar time-in-rank (14.7 years for men and 15.0 years for women). The biggest pay gap by gender we see when looking at MD and PhD salaries separately is among MDs at the Associate Professor level, where women’s salaries are 84% of men’s (Fig. 3a; $200,987 compared with $238,653, mean time-in-rank for men = 10.6 years, for women = 10.1 years). While some of the variations seen may be due to small numbers or differences in time-in-rank (expecting people in a rank longer to be earning higher salaries), this gap warrants further study.

## Revenues and Roles in Departmental Activities

**Fig. 4:**
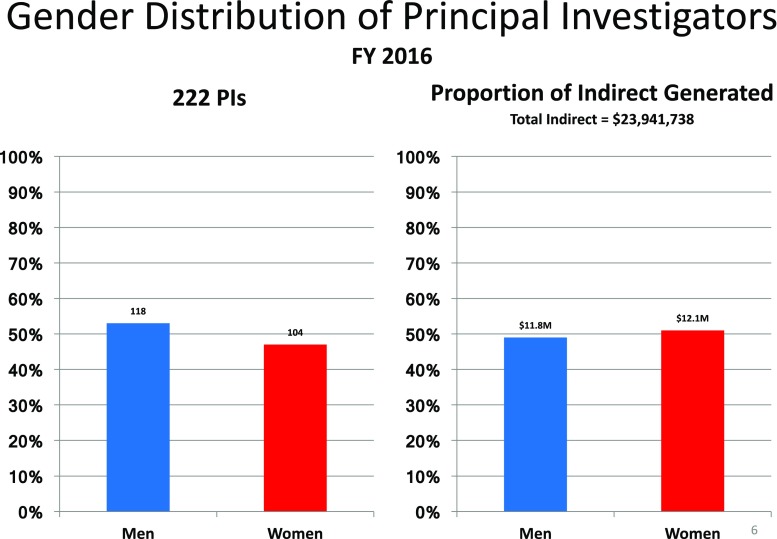
Gender distribution of Columbia Psychiatry Principal Investigators on grants and contracts awarded in Fiscal Year 2016

The two major sources of revenue generation in academic departments are through sponsored research (research productivity is the gold standard by which faculty are evaluated in academic medicine) and clinical services. Sponsored research, particularly awards funded by the NIH, is prestigious and important source of revenue for faculty, the department, and university, because of the indirect costs associated with them. In 2016, there were 222 Columbia Psychiatry faculty with active sponsored research that generated indirect cost revenues of $24 million. These funds are critical to the university and department’s functioning. Forty-seven percent of these 222 individuals were women, and, in the aggregate, women faculty generated 51% of the total annual indirect cost revenue (Fig. 4).

Women faculty generated a larger proportion of the aggregate clinical revenues than men faculty 57 vs 43%), but less per FTE. However, these differences were principally due to the fact that there are more women than men Ph.D. faculty, and the fees for services rendered by Ph.D. faculty typically are less than those of M.D. faculty.

Endowed chairs represent a precious and prestigious departmental resource. While endowed chairs are often established from donations for a specific person or area of expertise, there is often discretionary authority associated with their allocation. Endowed chairs typically underwrite a minimum of $100,000 toward the salary of the faculty member holding the endowed chair, in addition to any other benefits associated with the chair. A decade ago, only one of the department’s 9 endowed chairs (11%) was held by a woman. Since then, the department has acquired 9 additional endowed chairs, and 6 of the department’s 18 chairs (33%) are held by women.

The department supports a weekly grand rounds program 11 months of the year, to which it invites speakers with various expertise on mental health related topics. We examined the gender distribution of grand rounds speakers and found that, historically, men have predominated as grand rounds speakers, particularly for the named lectureships that come with larger honoraria and travel budgets. Over the past decade, roughly one third of grand rounds speakers have been women. When we shared these statistics with the individuals responsible for identifying and inviting speakers for grand rounds, this simple act of focusing attention on the gender of speakers stimulated an immediate course correction for gender distribution of grand rounds speakers.

## Challenges and Next Steps

Columbia Psychiatry has made significant progress with respect to gender equity. At the same time, we also see the need to sustain and improve our efforts. Like other academic departments and scientific organizations that have engaged in such self-examination, we have found that it is possible to make substantive improvements in supporting the career development of women faculty and that, at least for the present, ongoing monitoring and intervention appears essential [11–13]. We were unable to obtain comparable data from other departments within or outside of our university to use as benchmarks. The AAMC’s annual survey of member institution collects data on salary and gender [10]; reporting salary data by gender as part of the widely circulated annual AAMC Faculty Salary Report would make such benchmarking readily available.

Many of the concerns that prompted us to look at gender and academic standing also exist with respect to under-represented minorities. Expanding this exercise by surveying the proportions of under-represented minorities may also shine spotlights on progress, gaps, and barriers there. Here again, because the numbers at any given institution can be small, data aggregated by AAMC or other national resources would be helpful.

We firmly believe that identifying and addressing inequities in career development improves departments. Our emphasis has been to measure, monitor, and offer support where support seems needed, such as ensuring supportive mentoring, establishing a women’s faculty support group and providing a dedicated room for use by nursing rooms. Even if only some faculty benefit from these initiatives, they have a valuable impact and send a strong message that the department administration if trying to help all people advance their careers. Failure to do so runs the risk of losing talented individuals at vulnerable stages of their career development.

Achieving gender parity entails a commitment to gender parity, including an ongoing sensitivity to the issue and willingness to be proactive in addressing barriers, biases and establishing policies and procedures designed to serve this important goal. For example, because women on average may be less assertive in seeking career advancement [14], establishing objective reviews of the types of data offered above may help combat bias by alerting department administration to potential problems and prompting efforts to combat unfavorable trends [2]. Women may lack mentors (of either gender) or women role models, hence the importance of finding ways to reward the time spent in mentorship and to developing policies and mechanisms for career development and guidance. Administrative procedures also should help ensure representation of women in visible department activities such as grand rounds, visiting professorships, and committee membership. Work-life issues such as child/elder care and finding positions geographically close to a spouse or partner often disproportionately affect women [2]; hence, it is important to look for ways to support flexibility and encouraging faculty to ask to see what might be possible.

In 2006, we initiated a Women Faculty Group for the purpose of helping women faculty develop their careers and address gender-specific challenges often encountered by women. As noted in the findings reported above, gender disparities were greater a decade ago than they are today, and the group has evolved as the department evolved, with different generations of women leading the initiative. Today, all faculty and trainees (men and women) are welcome at the monthly Women Faculty Group meetings. In addition to the networking opportunities provided, monthly meetings have included skills training workshops plus talks by senior women at Columbia and elsewhere who have recounted the joys and challenges that comprise their professional biographies. Popular themes at these meetings have included getting a seat at the table, negotiating deals, routes to work-life balance, and dealing with difficult employees, usually as part of “how I got here” presentations from women department heads and vice deans at Columbia. In addition to the information conveyed, these meetings help insure that people see senior women role models, whether they come to the meetings or simply see the talks announced in the weekly bulletin. The Department Chair also has met regularly with the WFG group as a means of reviewing progress, hearing concerns, and being proactive.

Perhaps most important procedurally is to keep monitoring the data on departmental investments by gender to be sure that we can identify trends over time to identify which apparent discrepancies are real, identifying and addressing barriers along the way, and monitoring the results to determine if the corrective actions have the desired impact.

At Columbia Psychiatry, we have made a commitment to diversity and parity. The date in this paper reflects the progress that we have made with respect to gender parity. For ethical reasons, financial benefit, and to advance our mission, it is important to understand, monitor, establish mechanisms and commit the resources necessary to ensure that all faculty have opportunities for career advancement and success. This kind of close scrutiny of access, performance, and resources by gender is a potential model for other programs seeking to identify and address potential issues of gender disparity within their own departments. This process can also be applied to other faculty constituencies who may be subject to underrepresentation. By pursuing equal access, support and opportunity of all faculty, we can best reap the benefits of the intellectual resources of our population.
